# Supplementary material for: A Systematic Review and Meta-Analysis of Serologic Response following Coronavirus Disease 2019 (COVID-19) Vaccination in Solid Organ Transplant Recipients
Source: Viruses. 2022 Aug 19;14(8):1822. doi: 10.3390/v14081822 (PMC9413038; doi:10.3390/v14081822)
Supplement: Supplementary file 1 [file viruses-14-01822-s001.zip › viruses-1842260-supplementary.pdf]

Supplementary Figure S1.

A. Serologic response after one dose of vaccine: Ad26.COV2.S vaccine

Study	Event rate	Lower limit	Upper limit	Total	Relative weight
Boyarsky 3 (Ad26.COV2.S/Mixed)	0.167	0.042	0.477	2 / 12	100.00
**Overall**	**0.167**	**0.042**	**0.477**	**2 / 12**	

Event rate and 95% CI

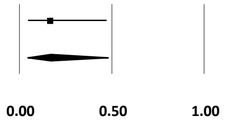

Overall: Heterogeneity: I^2 = 0%, Q = 0, P = 1.00

Supplementary Figure S2.

A. Funnel plot of studies included in meta-analysis of serologic response after one dose of vaccine

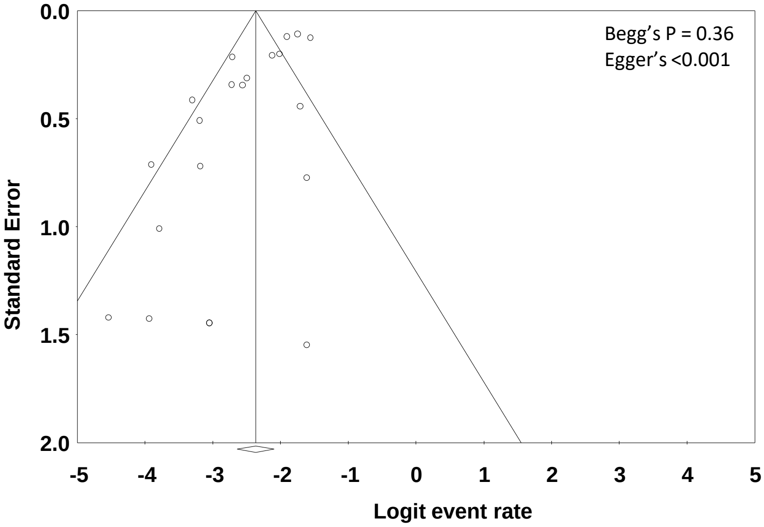

B. Sensitivity analysis excluding one study at a time for serologic response after one dose of vaccine

Removed study	Point	Lower limit	Upper limit
Boyarsky 1	0.078	0.060	0.101
Wadei	0.084	0.064	0.108
Benotmane 1	0.081	0.061	0.107
Yi	0.086	0.066	0.111
Marion	0.088	0.068	0.113
Havlin	0.086	0.067	0.110
Shostak	0.090	0.070	0.115
Bertrand	0.086	0.067	0.111
Boyarsky 2	0.077	0.058	0.103
Mazzola	0.085	0.065	0.110
Firket	0.085	0.065	0.109
Chavarot 1	0.089	0.069	0.113
Ou	0.078	0.058	0.104
Kamar	0.088	0.068	0.113
Hallett	0.080	0.061	0.106
Stumpf	0.085	0.065	0.110
Zadok	0.081	0.062	0.105
Schramm	0.086	0.067	0.111
Westhoff	0.085	0.065	0.109
Schrezenmeier	0.085	0.066	0.110
**Overall**	**0.084**	**0.065**	**0.108**

Event rate (95% CI) with study removed

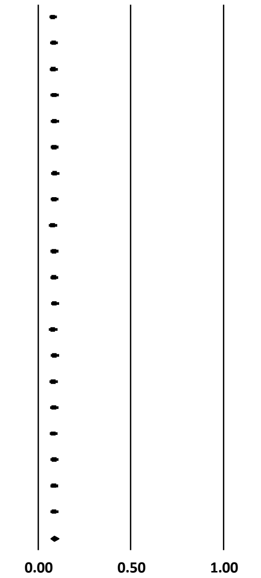

Supplementary Figure S2.

C. Sensitivity analysis according to type of mRNA vaccine for serologic response after one dose of vaccine

Subgroup	Study	Transplant	Event rate	Lower limit	Upper limit	Total	Relative weight
**BNT162b2**	Havlin	Heart/Lung	0.011	0.001	0.149	0 / 46	2.00
	Shostak	Heart/Lung	0.036	0.016	0.077	6 / 168	14.31
	Bertrand	Kidney	0.022	0.003	0.142	1 / 45	3.74
	Mazzola	Mix	0.072	0.038	0.133	9 / 125	17.37
	Firket	Kidney	0.045	0.003	0.448	0 / 10	1.94
	Chavarot 1	Kidney	0.020	0.005	0.076	2 / 101	6.75
	Kamar	Mix	0.040	0.015	0.101	4 / 101	11.08
	Stumpf	Kidney	0.076	0.043	0.133	11 / 144	19.01
	Zadok	Heart/Lung	0.154	0.071	0.303	6 / 39	13.24
	Schramm	Heart/Lung	0.040	0.010	0.146	2 / 50	6.63
	Westhoff	Kidney	0.045	0.003	0.448	0 / 10	1.94
	Schrezenmeier	Kidney	0.019	0.001	0.244	0 / 25	1.99
	**Subtotal**		**0.054**	**0.036**	**0.078**	**41 / 864**	
**Mixed**	Boyarsky 1	Mix	0.174	0.142	0.213	76 / 436	18.89
	Wadei	Mix	0.167	0.010	0.806	0 / 2	0.96
	Yi	Kidney	0.062	0.033	0.115	9 / 145	10.50
	Marion	Mix	0.063	0.042	0.093	23 / 367	15.27
	Boyarsky 2	Mix	0.149	0.124	0.178	98 / 658	19.49
	Ou	Mix	0.130	0.105	0.160	77 / 592	19.04
	Hallett	Heart/Lung	0.118	0.083	0.166	28 / 237	15.85
	**Subtotal**		**0.116**	**0.088**	**0.152**	**311 / 2437**	
**mRNA-1273**	Benotmane 1	Kidney	0.107	0.074	0.153	26 / 242	100.00
	**Subtotal**		**0.107**	**0.074**	**0.153**	**26 / 242**	
**Overall**			**0.093**	**0.077**	**0.113**	**378 / 3543**	

Event rate and 95% CI

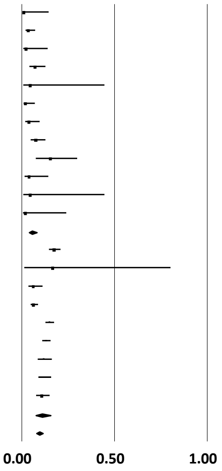

BNT162b2 : Heterogeneity: I^2 = 28.12%, Q = 15.30 P = 0.17
Mixed: Heterogeneity: I^2 = 79.45%, Q = 29.20, P <0.001
mRNA-1273 : Heterogeneity: I^2 = 0%, Q = 0, P = 1.00
Overall: Heterogeneity: I^2 = 74.17%, Q = 73.55, P <0.001

Supplementary Figure S3.

A. Funnel plot of studies included in meta-analysis of serologic response after two doses of vaccine

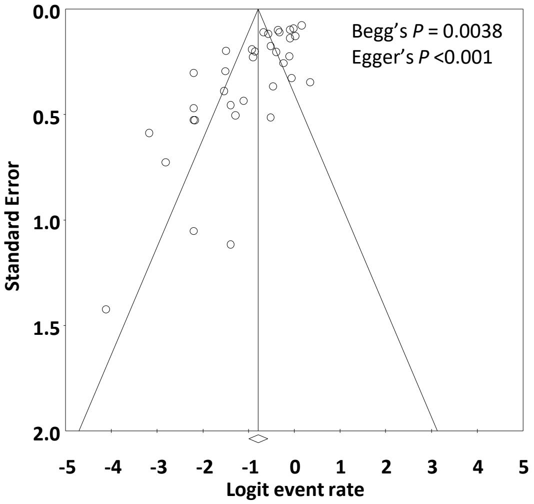

B. Sensitivity analysis excluding one study at a time for serologic response after two doses of vaccine

Removed study name	Point	Lower limit	Upper limit
Grupper	0.309	0.266	0.356
Wadei	0.313	0.271	0.359
Rincon-Arevalo	0.319	0.277	0.365
Benotmane 2	0.305	0.262	0.352
Rabinowich	0.306	0.264	0.353
Peled 1	0.318	0.276	0.364
Sattler	0.319	0.277	0.365
Marion	0.310	0.266	0.357
Miele	0.311	0.268	0.357
Havlin	0.316	0.274	0.362
Rosen-Zvi	0.309	0.265	0.356
Shostak	0.320	0.278	0.365
Bertrand	0.317	0.274	0.363
Cucchiari	0.313	0.269	0.359
Husain	0.314	0.271	0.360
Korth	0.315	0.272	0.361
Boyarsky 2	0.305	0.263	0.350
Mazzola	0.313	0.270	0.360
Firket	0.315	0.272	0.360
Danthu	0.323	0.281	0.368
Marinaki	0.305	0.263	0.351
Chavarot 1	0.319	0.277	0.365
Ou	0.304	0.261	0.352
Kamar	0.308	0.265	0.355
Hallett	0.305	0.262	0.351
Stumpf	0.306	0.262	0.354
Zadok	0.307	0.265	0.353
Schramm	0.320	0.278	0.366
Werbel	0.315	0.273	0.362
Hall	0.325	0.283	0.370
Karaba	0.310	0.267	0.356
Masset	0.304	0.261	0.351
Del Bello	0.306	0.262	0.354
Charmetant	0.313	0.270	0.359
Massa	0.308	0.265	0.354
**Overall**	**0.312**	**0.270**	**0.358**

Event rate (95% CI) with study removed

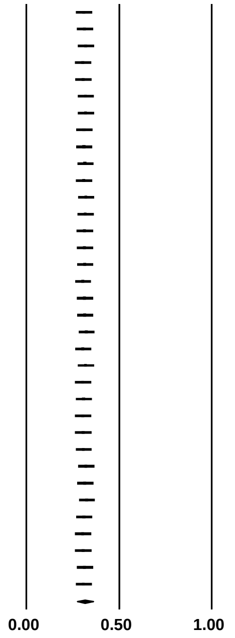

Supplementary Figure S3.

C. Sensitivity analysis excluding preprint studies for serologic response after two dose of vaccine

Subgroup	Study	Event rate	Lower limit	Upper limit	Total	Relative weight
**Heart/Lung**	Peled 1	0.182	0.111	0.284	14 / 77	18.74
	Havlin	0.016	0.001	0.211	0 / 30	6.56
	Shostak	0.185	0.133	0.250	31 / 168	19.63
	Hallett	0.506	0.443	0.570	120 / 237	20.09
	Zadok	0.486	0.332	0.644	18 / 37	18.37
	Schramm	0.100	0.042	0.219	5 / 50	16.60
	**Subtotal**	**0.229**	**0.111**	**0.415**	**188 / 599**	
**Kidney**	Grupper	0.375	0.298	0.459	51 / 136	9.66
	Rincon-Arevalo	0.100	0.038	0.238	4 / 40	5.33
	Benotmane 2	0.478	0.410	0.546	98 / 205	10.05
	Sattler	0.103	0.039	0.243	4 / 39	5.32
	Rosen-Zvi	0.364	0.312	0.419	112 / 308	10.24
	Bertrand	0.178	0.091	0.317	8 / 45	6.91
	Cucchiari	0.299	0.223	0.388	35 / 117	9.37
	Husain	0.250	0.124	0.439	7 / 28	6.33
	Korth	0.217	0.093	0.428	5 / 23	5.55
	Firket	0.100	0.014	0.467	1 / 10	2.12
	Danthu	0.041	0.013	0.118	3 / 74	4.73
	Chavarot 1	0.057	0.014	0.202	2 / 35	3.65
	Stumpf	0.420	0.369	0.474	140 / 333	10.31
	Masset	0.498	0.452	0.544	227 / 456	10.44
	**Subtotal**	**0.268**	**0.207**	**0.338**	**697 / 1849**	
**Liver**	Rabinowich	0.475	0.368	0.584	38 / 80	100.00
	**Subtotal**	0.475	0.368	0.584	38 / 80	
**Mix**	Wadei	0.200	0.027	0.691	1 / 5	2.00
	Marion	0.338	0.291	0.388	124 / 367	11.71
	Miele	0.375	0.179	0.623	6 / 16	5.85
	Boyarsky 2	0.543	0.504	0.580	357 / 658	11.99
	Mazzola	0.286	0.215	0.368	38 / 133	10.67
	Marinaki	0.588	0.419	0.739	20 / 34	8.19
	Ou	0.478	0.429	0.527	191 / 400	11.81
	Kamar	0.404	0.312	0.503	40 / 99	10.48
	Werbel	0.200	0.093	0.379	6 / 30	6.61
	Hall	0.100	0.058	0.168	12 / 120	8.90
	Del Bello	0.414	0.367	0.463	164 / 396	11.79
	**Subtotal**	**0.363**	**0.289**	**0.444**	**959 / 2258**	
**Overall**		**0.340**	**0.296**	**0.387**	**1882 / 4786**	

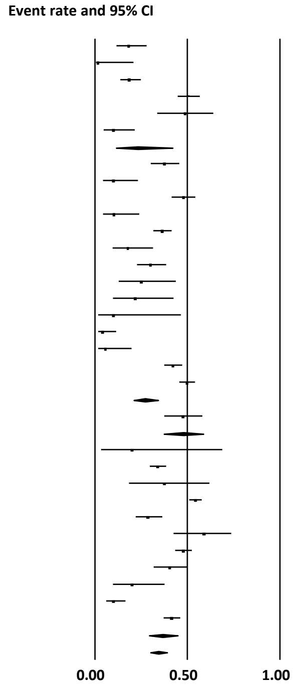

Heart/Lung: Heterogeneity: I^2 = 93.12%, Q = 72.67, P <0.001
Kidney: Heterogeneity: I^2 = 87.36%, Q = 102.82, P <0.001
Liver: Heterogeneity: I^2 = 0%, Q = 0, P = 1.00
Mixed: Heterogeneity: I^2 = 90.99%, Q = 110.97, P <0.001
Overall: Heterogeneity: I^2 = 89.66%, Q = 299.82, P <0.001

Supplementary Figure S3.

D. Sensitivity analysis according to type of mRNA vaccine for serologic response after two doses of vaccine

Subgroup	Study	Transplant	Event rate	Lower limit	Upper limit	Total	Relative weight
BNT162b2	Grupper	Kidney	0.375	0.298	0.459	51 / 136	5.47
	Rincon-Arevalo	Kidney	0.100	0.038	0.238	4 / 40	3.07
	Rabinowich	Liver	0.475	0.368	0.584	38 / 80	5.16
	Peled 1	Heart/Lung	0.182	0.111	0.284	14 / 77	4.65
	Sattler	Kidney	0.103	0.039	0.243	4 / 39	3.07
	Miele	Mix	0.375	0.179	0.623	6 / 16	3.13
	Havlin	Heart/Lung	0.016	0.001	0.211	0 / 30	0.74
	Rosen-Zvi	Kidney	0.364	0.312	0.419	112 / 308	5.79
	Shostak	Heart/Lung	0.185	0.133	0.250	31 / 168	5.33
	Bertrand	Kidney	0.178	0.091	0.317	8 / 45	3.96
	Korth	Kidney	0.217	0.093	0.428	5 / 23	3.20
	Mazzola	Mix	0.286	0.215	0.368	38 / 133	5.38
	Firket	Kidney	0.100	0.014	0.467	1 / 10	1.24
	Danthu	Kidney	0.041	0.013	0.118	3 / 74	2.73
	Marinaki	Mix	0.588	0.419	0.739	20 / 34	4.25
	Chavarot 1	Kidney	0.057	0.014	0.202	2 / 35	2.12
	Kamar	Mix	0.404	0.312	0.503	40 / 99	5.29
	Stumpf	Kidney	0.420	0.369	0.474	140 / 333	5.82
	Zadok	Heart/Lung	0.486	0.332	0.644	18 / 37	4.40
	Schramm	Heart/Lung	0.100	0.042	0.219	5 / 50	3.41
	Masset	Kidney	0.498	0.452	0.544	227 / 456	5.89
	Del Bello	Mix	0.414	0.367	0.463	164 / 396	5.86
	Charmetant	Kidney	0.290	0.207	0.390	27 / 93	5.13
	Massa	Kidney	0.443	0.324	0.568	27 / 61	4.92
	**Subtotal**		**0.289**	**0.239**	**0.345**	**985 / 2773**	
Mixed	Wadei	Mix	0.200	0.027	0.691	1 / 5	2.10
	Marion	Mix	0.338	0.291	0.388	124 / 367	17.68
	Husain	Kidney	0.250	0.124	0.439	7 / 28	8.54
	Boyarsky 2	Mix	0.543	0.504	0.580	357 / 658	18.34
	Ou	Mix	0.478	0.429	0.527	191 / 400	17.91
	Hallett	Heart/Lung	0.506	0.443	0.570	120 / 237	17.19
	Werbel	Mix	0.200	0.093	0.379	6 / 30	8.12
	Karaba	Mix	0.387	0.235	0.565	12 / 31	10.14
	**Subtotal**		**0.406**	**0.328**	**0.489**	**818 / 1756**	
mRNA-1273	Benotmane 2	Kidney	0.478	0.410	0.546	98 / 205	34.51
	Cucchiari	Kidney	0.299	0.223	0.388	35 / 117	33.68
	Hall	Mix	0.100	0.058	0.168	12 / 120	31.81
	**Subtotal**		**0.266**	**0.110**	**0.513**	**145 / 442**	
**Overall**			**0.328**	**0.285**	**0.373**	**1948 / 4971**	

Event rate and 95% CI

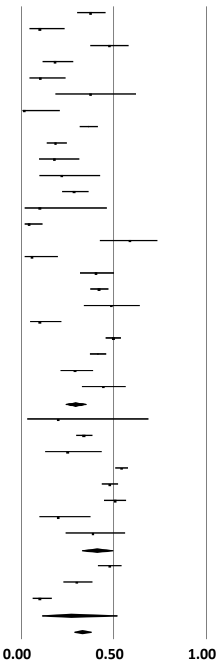

BNT162b2 : Heterogeneity: I^2 = 86.55%, Q = 170.97 P <0.001
Mixed: Heterogeneity: I^2 = 87.29%, Q = 55.08, P <0.001
mRNA-1273 : Heterogeneity: I^2 = 95.29%, Q = 42.43, P <0.001
Overall: Heterogeneity: I^2 = 88.88%, Q = 305.80, P <0.001

Supplementary Figure S4.

A. Funnel plot of studies included in meta-analysis of comparison of serologic response after one dose of vaccine compared to controls

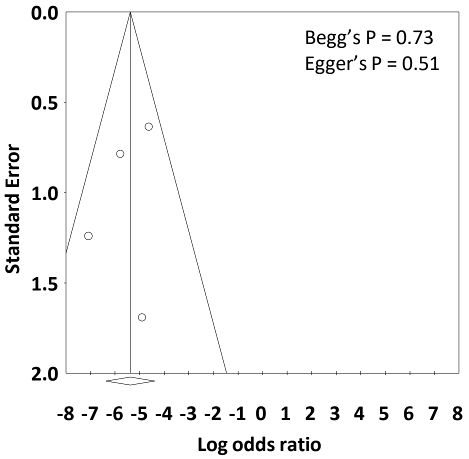

B. Funnel plot of studies included in meta-analysis of comparison of serologic response after two doses of vaccine compared to controls

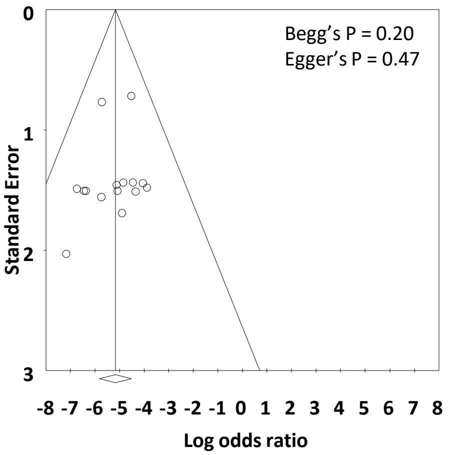

Supplementary Figure S5.

A. Funnel plot of studies included in meta-analysis of serologic response after three doses of vaccine

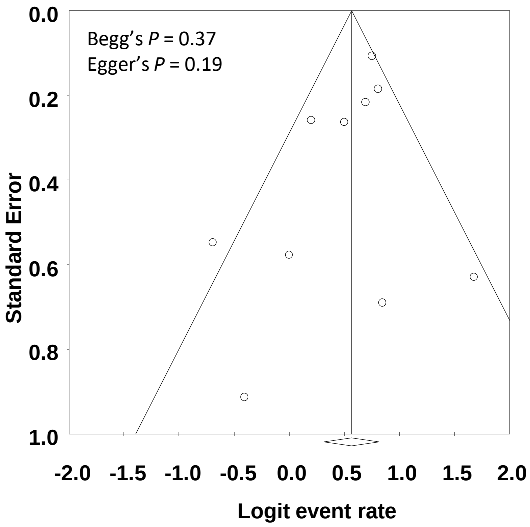

B. Sensitivity analysis according to type of vaccine for serologic response after three doses of vaccine

Subgroup	Study	Transplant	Event rate	Lower limit	Upper limit	Total	Relative weight
**Different mRNA**	Werbel BNT162b2	Mix	0.400	0.100	0.800	2 / 5	25.39
	Werbel mRNA-1273	Mix	0.700	0.376	0.900	7 / 10	35.55
	Karaba mRNA	Mix	0.842	0.608	0.948	16 / 19	39.07
	**Subtotal**		**0.701**	**0.437**	**0.876**	**25 / 34**	
**Same mRNA**	Hall	Mix	0.550	0.424	0.670	33 / 60	10.90
	Peled 2	Heart/Lung	0.667	0.567	0.754	64 / 96	15.06
	Masset	Kidney	0.691	0.609	0.763	94 / 136	19.59
	Del Bello	Mix	0.679	0.632	0.723	269 / 396	43.89
	Massa	Kidney	0.623	0.496	0.735	38 / 61	10.55
	**Subtotal**		**0.661**	**0.620**	**0.699**	**498 / 749**	
**mRNA to AD26**	Werbel AD26.COV2.S	Mix	0.333	0.146	0.594	5 / 15	52.63
	Karaba AD26.COV2.S	Mix	0.500	0.244	0.756	6 / 12	47.37
	**Subtotal**		**0.410**	**0.242**	**0.602**	**11 / 27**	
**Overall**			**0.651**	**0.611**	**0.688**	**534 / 810**	

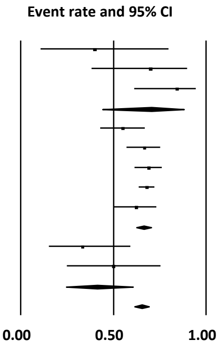

Different mRNA: Heterogeneity: I^2 = 43.68%, Q = 3.55, P = 0.17
Same mRNA: Heterogeneity: I^2 = 15.89%, Q = 4.76, P = 0.31
mRNA to AD26.COV2.S: Heterogeneity: I^2 = 0%, Q = 0.76, P = 0.38
Overall: Heterogeneity: I^2 = 44.69%, Q = 16.27, P = 0.061

C. Sensitivity analysis excluding one study at a time for serologic response after three doses of vaccine

Study	Point	Lower limit	Upper limit
Werbel BNT162b2	0.642	0.583	0.697
Werbel mRNA-1273	0.634	0.571	0.693
Werbel AD26.COV2.S	0.657	0.612	0.699
Hall	0.652	0.593	0.707
Karaba mRNA	0.631	0.574	0.685
Karaba AD26.COV2.S	0.643	0.583	0.700
Peled 2	0.628	0.556	0.695
Masset	0.622	0.549	0.690
Del Bello	0.621	0.543	0.693
Massa	0.637	0.569	0.701
	0.638	0.578	0.693

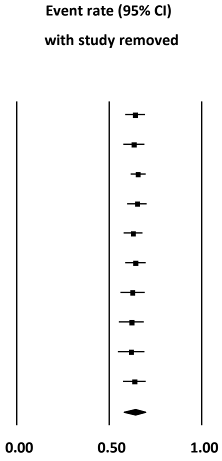

Supplementary Figure S6.

A. Funnel plot of studies included in meta-analysis of serologic response after three doses of vaccine among non-responders to two doses

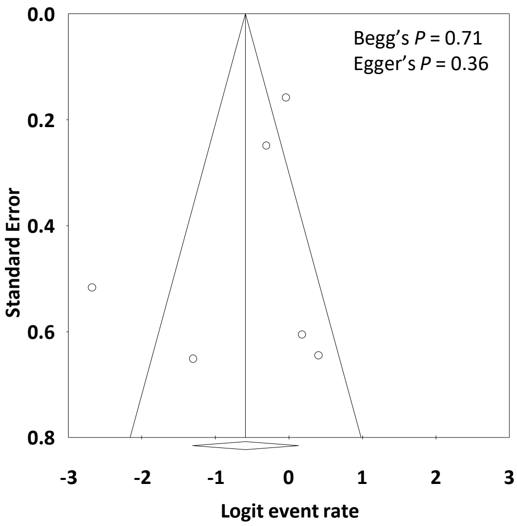

B. Sensitivity analysis according to type of vaccine for serologic response after three doses of vaccine among non-responders to two doses

Subgroup	Study	Transplant	Event rate	Lower limit	Upper limit	Total	Relative weight
**Same mRNA**	Benotmane 3	Kidney	0.491	0.414	0.568	78 / 159	29.75
	Charmetant	Kidney	0.424	0.311	0.546	28 / 66	28.35
	Chavarot 2	Kidney	0.065	0.024	0.160	4 / 62	22.49
	Schrezenmeier BNT 162b2	Kidney	0.214	0.071	0.494	3 / 14	19.41
	**Subtotal**		**0.279**	**0.133**	**0.494**	**113 / 301**	
**BNT162b2 to AZD1222**	Schrezenmeier AZD1222	Kidney	0.545	0.268	0.797	6 / 11	100.00
	**Subtotal**		**0.545**	**0.268**	**0.797**	**6 / 11**	
**BNT162b2 to mRNA-1273**	Westhoff	Kidney	0.600	0.297	0.842	6 / 10	100.00
	**Subtotal**		**0.600**	**0.297**	**0.842**	**6 / 10**	
**Overall**			**0.428**	**0.284**	**0.585**	**125 / 322**	

Event rate and 95% CI

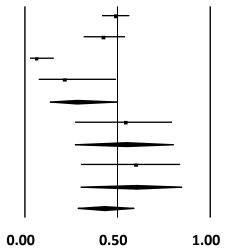

Same mRNA: Heterogeneity: I^2 = 88.54%, Q = 26.17, P <0.001
BNT162b2 to AZD1222 : Heterogeneity: I^2 = 0%, Q = , P = 1.00
BNT162b2 to mRNA-1273: Heterogeneity: I^2 = 0%, Q = , P = 1.00
Overall: Heterogeneity: I^2 = 82.11%, Q = 27.94, P <0.001

C. Sensitivity analysis excluding one study at a time for serologic response after three doses of vaccine among non-responders to two doses

Study	Point	Lower limit	Upper limit
Benotmane 3	0.322	0.143	0.574
Charmetant	0.337	0.147	0.599
Chavarot 2	0.462	0.383	0.543
Westhoff	0.321	0.175	0.513
Schrezenmeier BNT 162b2	0.382	0.221	0.574
Schrezenmeier AZD1222	0.326	0.176	0.523
	0.357	0.212	0.533

Event rate (95% CI) with study removed

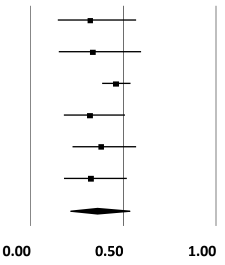

Supplementary Table S1. Risk of bias assessment by Joanna Briggs Institute Critical Appraisal Checklist

Author	Year	Q1	Q2	Q3	Q4	Q5	Q6	Q7	Q8	Q9
1. **Grupper**	2021	Yes	Yes	Yes	Yes	No	Yes	Yes	Yes	Yes
2. **Boyarsky**	2021	Yes	Yes	Yes	No	No	NA	No	Yes	Yes
3. **Wadei**	2021	Yes	Yes	Yes	No	No	Yes	No	Unclear	Yes
4. **Rincon-Arevalo**	2021	Yes	Yes	Yes	Yes	Yes	Yes	Yes	Yes	Yes
5. **Benotmane**	2021	Yes	Yes	Yes	No	No	Yes	Yes	Yes	Yes
6. **Benotmane**	2021	Yes	Yes	Yes	No	Yes	Yes	Yes	Yes	Yes
7. **Rabinowich**	2021	Yes	Yes	Yes	Yes	No	Yes	Yes	Yes	Yes
8. **Yi**	2021	Yes	Yes	Unclear	Yes	Yes	Yes	Yes	Yes	Yes
9. **Peled**	2021	Yes	Yes	Yes	Yes	No	Yes	Yes	Yes	Yes
10. **Sattler**	2021	Yes	Yes	Yes	Yes	Yes	Yes	Yes	Yes	Yes
11. **Marion**	2021	Yes	Yes	Yes	No	Yes	No	No	Yes	Yes
12. **Miele**	2021	Yes	Yes	Yes	Yes	No	Yes	Yes	Yes	Yes
13. **Havlin**	2021	Yes	Yes	Yes	Yes	Yes	Yes	Yes	Yes	Yes
14. **Rosen-Zvi**	2021	Yes	Yes	Yes	No	No	Yes	Yes	Yes	Yes
15. **Shostak**	2021	Yes	Yes	Yes	No	No	Yes	Yes	Yes	Yes
16. **Bertrand**	2021	Yes	Yes	Yes	No	Yes	Yes	Yes	Yes	Yes
17. **Cucchiari**	2021	Yes	Yes	Yes	No	Yes	Yes	Yes	Yes	Yes
18. **Husain**	2021	Yes	Yes	Yes	No	No	Yes	No	Yes	Yes
19. **Korth**	2021	Yes	Yes	Yes	Yes	No	Yes	Yes	Yes	Yes
20. **Boyarsky**	2021	Yes	Yes	Yes	No	No	Yes	No	Yes	Yes
21. **Mazzola**	2021	Yes	Yes	Yes	Yes	Yes	No	Yes	Yes	Yes
22. **Firket**	2021	Yes	Yes	Yes	Yes	Yes	Yes	Yes	Yes	Yes
23. **Danthu**	2021	Yes	Yes	Yes	Yes	Yes	Yes	Yes	Yes	Yes
24. **Boyarsky**	2021	Yes	Yes	Yes	No	No	Yes	Yes	Yes	Yes
25. **Marinaki**	2021	Yes	Yes	Yes	Yes	No	Yes	Yes	Yes	Yes
26. **Chavarot**	2021	Yes	Yes	Yes	No	No	Yes	No	Yes	Yes
27. **Ou**	2021	Yes	Yes	Yes	No	No	No	No	Yes	Yes
28. **Kamar**	2021	Yes	Yes	Yes	No	Yes	No	Yes	Yes	Yes
29. **Hallett**	2021	Yes	Yes	Yes	No	No	No	No	Yes	Yes
30. **Stumpf**	2021	Yes	Yes	Yes	Yes	No	No	No	Yes	Yes
31. **Zadok**	2021	Yes	Yes	Yes	No	No	No	No	Yes	Yes
32. **Schramm**	2021	Yes	Yes	Yes	Yes	No	No	No	Yes	Yes
33. **Werbel**	2021	Yes	Yes	Yes	No	Yes	No	No	Yes	Unclear
34. **Hall**	2021	Yes	Yes	Yes	Unclear	Yes	Yes	Yes	Yes	Yes
35. **Karaba**	2021	Yes	Yes	Yes	Unclear	No	Yes	Yes	Yes	Yes
36. **Peled**	2021	Yes	Yes	Yes	No	Yes	Yes	Yes	Yes	Yes
37. **Benotmane**	2021	Yes	Yes	Yes	No	Yes	Yes	Yes	Yes	Yes
38. **Masset**	2021	Yes	Yes	Yes	No	Yes	Yes	No	Yes	Unclear
39. **Del Bello**	2021	Yes	Yes	Yes	No	Yes	Yes	No	Yes	Yes
40. **Charmetant**	2021	Yes	Yes	Yes	No	Yes	Yes	Yes	Yes	Yes
41. **Chavarot**	2021	Yes	Yes	Yes	No	Yes	Yes	Yes	Yes	Yes

**42.**	**Westhoff**	2021	Yes	Yes	Yes	No	Yes	Yes	Yes	Yes	Yes
**43.**	**Massa**	2021	Yes	Yes	Yes	No	Yes	Yes	Yes	Yes	Yes
**44.**	**Schrezenmeier**	2021	Yes	Yes	Yes	No	Yes	Yes	Yes	Yes	Yes

Q1: Is it clear in the study what is the ‘cause’ and what is the ‘effect’ (i.e. there is no confusion about which variable comes first)?; Q2: Were the participants included in any comparisons similar?; Q3: Were the participants included in any comparisons receiving similar treatment/care, other than the exposure or intervention of interest?; Q4: Was there a control group?; Q5: Were there multiple measurements of the outcome both pre and post the intervention/exposure?; Q6: Was follow up complete and if not, were differences between groups in terms of their follow up adequately described and analyzed?; Q7: Were the outcomes of participants included in any comparisons measured in the same way?; Q8: Were outcomes measured in a reliable way?; Q9: Was appropriate statistical analysis used?; NA not applicable.

Supplementary Table S2. Univariate and multivariate meta-regression models of variables associated with serologic response after one dose of mRNA vaccine

	**Univariate meta-regression**			**Multivariate meta-regression**		
	**Coefficient**	**95% CI**	**P-value**	**Coefficient**	**95% CI**	**P-value**
**Age**	-0.036	-0.16-0.084	0.56	-0.050	-0.18-0.079	0.45
**Female**	0.025	0.010-0.040	0.0010	-0.011	-0.042-0.021	0.51
**Steroid**	-0.025	-0.044-(-0.0050)	0.014	-0.029	-0.052-(-0.0061)	**0.013**
**BNT162b2 vs. Mixed**	0.84	0.33-1.34	0.0011	0.58	-0.41-1.57	0.25
**BNT162b2 vs. mRNA-1273**	0.75	-0.13-1.62	0.094	0.74	-0.15-1.62	0.10
**Heart/lung vs. Kidney**	-0.23	-0.98-0.52	0.55	-0.80	-1.81-0.22	0.12
**Heart/lung vs. Mixed**	0.35	-0.33-1.03	0.31	-0.13	-0.91-0.64	0.74

Supplementary Table S3. Univariate and multivariate meta-regression models of variables associated with serologic response after two doses of mRNA vaccine

	**Univariate meta-regression**			**Multivariate meta-regression**		
	**Coefficient**	**95% CI**	**P-value**	**Coefficient**	**95% CI**	**P-value**
**Age**	-0.047	-0.12-0.026	0.21	-0.10	-0.19-(-0.020)	**0.016**
**Female**	0.0013	-0.015-0.017	0.87	-0.019	-0.043-0.0046	0.12
**Steroid**	-0.0096	-0.018-(-0.0015)	0.021	-0.014	-0.025-(-0.0024)	**0.017**
**BNT162b2 vs. Mixed**	0.46	-0.030-0.95	0.066	0.52	0.25-1.29	0.19
**BNT162b2 vs. mRNA-1273**	-0.074	-0.76-0.61	0.83	0.23	-0.58-1.04	0.58
**Heart/lung vs. Kidney**	0.10	-0.52-0.73	0.75	-0.39	-1.23-0.46	0.37
**Heart/lung vs. Liver**	0.96	-0.32-2.24	0.14	-0.0008	-1.52-1.52	1.00
**Heart/lung vs. Mixed**	0.49	-0.15-1.14	.13	-0.18	-1.02-0.65	0.67

Supplementary Table S4. Grading of Recommendations, Assessment, Development, and Evaluation (GRADE) criteria for studies included in the meta-analysis

(A) Meta-analysis of observational studies assessing serological response after one dose of vaccine

Number of participants	Starting Level of Evidence	Quality assessment					Reasons to increase level of evidence (Large magnitude of effect; Dose-response gradient; Potential confounding)	Overall quality of evidence
		Risk of Bias	Inconsistency	Indirectness	Imprecision	Publication bias		
3543	Low	Not serious	Not serious	Serious	Not serious	Not serious	N/A	Low

(B) Meta-analysis of case control studies comparing serological response after one dose of vaccine to controls

Number of participants	Starting Level of Evidence	Quality assessment					Reasons to increase level of evidence (Large magnitude of effect; Dose-response gradient; Potential confounding)	Overall quality of evidence
		Risk of Bias	Inconsistency	Indirectness	Imprecision	Publication bias		
349 (cases) and 146 (controls)	Low	Not serious	Serious	Serious	Not serious	Not serious	N/A	Low

(C) Meta-analysis of observational studies assessing serological response after two doses of vaccine

Number of participants	Starting Level of Evidence	Quality assessment					Reasons to increase level of evidence (Large magnitude of effect; Dose-response gradient; Potential confounding)	Overall quality of evidence
		Risk of Bias	Inconsistency	Indirectness	Imprecision	Publication bias		
4971	Low	Not serious	Not serious	Serious	Not serious	Not serious	N/A	Low

(D) Meta-analysis of case control studies comparing serological response after two doses of vaccine to controls

Number of participants	Starting Level of Evidence	Quality assessment					Reasons to increase level of evidence (Large magnitude of effect; Dose-response gradient; Potential confounding)	Overall quality of evidence
		Risk of Bias	Inconsistency	Indirectness	Imprecision	Publication bias		
1106 (cases) and 673 (controls)	Low	Not serious	Serious	Serious	Not serious	Not serious	N/A	Low

(E) Meta-analysis of observational studies assessing serological response after three doses of vaccine

Number of participants	Starting Level of Evidence	Quality assessment					Reasons to increase level of evidence (Large magnitude of effect; Dose-response gradient; Potential confounding)	Overall quality of evidence
		Risk of Bias	Inconsistency	Indirectness	Imprecision	Publication bias		
810	Low	Not serious	Not serious	Serious	Not serious	Not serious	N/A	Low
